# Impact of N on the atomic-scale Sb distribution in quaternary GaAsSbN-capped InAs quantum dots

**DOI:** 10.1186/1556-276X-7-653

**Published:** 2012-11-27

**Authors:** Daniel F Reyes, David González, Jose M Ulloa, David L Sales, Lara Dominguez, Alvaro Mayoral, Adrian Hierro

**Affiliations:** 1 Departamento de Ciencia de los Materiales e IM y QI, Universidad de Cádiz, Puerto Real, Cádiz, 11510, Spain; 2 Institute for Systems based on Optoelectronics and Microtechnology (ISOM) and Departamento Ingenieria Electronica, Universidad Politecnica de Madrid, Ciudad Universitaria s/n, Madrid, 28040, Spain; 3 Laboratorio de Microscopías Avanzadas (LMA), Instituto de Nanociencia de Aragón(INA), Universidad de Zaragoza, Mariano Esquillor, Edificio I+D, Zaragoza, 50018, Spain

**Keywords:** III-V quantum dots, GaAsSb, N incorporation, Sb distribution, Strain state, 73.21.La quantum dots, 78.55.Cr III-V semiconductors, 68.55.Ln defects and impurities: doping, implantation, distribution, concentration, etc., 68.55.Nq composition and phase identification

## Abstract

The use of GaAsSbN capping layers on InAs/GaAs quantum dots (QDs) has recently been proposed for micro- and optoelectronic applications for their ability to independently tailor electron and hole confinement potentials. However, there is a lack of knowledge about the structural and compositional changes associated with the process of simultaneous Sb and N incorporation. In the present work, we have characterized using transmission electron microscopy techniques the effects of adding N in the GaAsSb/InAs/GaAs QD system. Firstly, strain maps of the regions away from the InAs QDs had revealed a huge reduction of the strain fields with the N incorporation but a higher inhomogeneity, which points to a composition modulation enhancement with the presence of Sb-rich and Sb-poor regions in the range of a few nanometers. On the other hand, the average strain in the QDs and surroundings is also similar in both cases. It could be explained by the accumulation of Sb above the QDs, compensating the tensile strain induced by the N incorporation together with an In-Ga intermixing inhibition. Indeed, compositional maps of column resolution from aberration-corrected Z-contrast images confirmed that the addition of N enhances the preferential deposition of Sb above the InAs QD, giving rise to an undulation of the growth front. As an outcome, the strong redshift in the photoluminescence spectrum of the GaAsSbN sample cannot be attributed only to the N-related reduction of the conduction band offset but also to an enhancement of the effect of Sb on the QD band structure.

## Background

A noteworthy effort has been made over the last years to broaden the emission wavelength of InAs/GaAs quantum dot (QD) lasers towards 1.55 μm, i.e., the telecommunication band which better matches the transmission characteristics of optical fibers, providing higher data rates at longer distances. The utilization of different types of capping layers (CL), such as strain-reducing layers (SRL) [1,2] or strain-compensating layers (SCL) [3], has been widely used to directly cover this issue by modifying the strain state of the QDs. Special attention has been paid to GaAsSb SRLs to take advantage of the surfactant role of Sb, which suppresses the defect generation and QD decomposition [4,5]. Additionally, GaAsSb capping layers present an extra degree of freedom as the emission band alignment changes from type I to type II at a certain Sb content [6]. A step forward is the addition of N in the CL to form a GaNAsSb quaternary system, which has been very recently realized [7]. As N reduces only the conduction band (CB) of GaAs and Sb rises the valence band (VB) of GaAs, the quaternary alloy GaAsSbN used as a CL for InAs/GaAs QDs allows independent tailoring of the electron and hole confinement potentials in a wide range, which could be useful for many applications [7].

However, there is a lack of knowledge about the structural and compositional changes associated with the process of simultaneous Sb and N incorporation. On one hand, the difficulty of incorporating antimony inside GaAs structures has repeatedly been reported in literature [8]. Indeed, the interdiffusion effects that affect the relative composition of In/Ga and As/Sb in and around the QD islands are still debated [9,10]. On the other hand, arising from specific properties of N such as its large electronegativity and small atomic volume, it is well known that dilute nitride alloys create statistically large compositional fluctuations [11].

The aim of this work is to describe, by advanced transmission electron microscopy (TEM) techniques, the atomic distribution in InAs QD capped by GaAsSb SRL with and without N and its effect on the photoluminescence spectra. The strain in the regions close and away from the InAs QDs is analyzed, allowing the estimation of the compositional distribution of the SRL and nanostructures. Moreover, we have qualitatively extracted chemical information from aberration-corrected Z-contrast images by calculation of integrated intensities of the atomic columns [12]. Our results reveal that the incorporation of N induces significant changes in the Sb distribution in the InAs/GaAsSb QD system.

## Methods

### Equipment and techniques

Two samples (S-Sb and S-SbN) were grown by solid-source molecular beam epitaxy on Si-doped (100) n+ GaAs substrates. The QDs in both samples were grown by depositing 2.7 monolayers (ML) of InAs at 450°C and 0.04 ML/s on an intrinsic GaAs buffer layer. A nominally 5.0-nm-thick GaAs_0.88_Sb_0.12_ layer grown at 470°C was used to cover the QDs in sample S-Sb, followed by 250 nm of GaAs. Sample S-SbN was identical, but a nominal 2% N content was added to the 5.0-nm-thick CL. This active N was generated from a radio frequency (RF) plasma source with a 0.1-sccm flow of pure N_2_ (6 N) and a RF power of 60 W. The photoluminescence (PL) was measured at 15 K using a closed-cycle helium cryostat and a He-Ne laser as the excitation source. The emitted light was dispersed through a 1-m spectrometer and detected with a liquid nitrogen-cooled Ge detector using standard lock-in techniques. Conventional transmission electron microscopy (CTEM) and high-resolution TEM (HRTEM) were carried out in a JEOL 2011 LaB_6_ filament microscope (JEOL Ltd., Akishima, Tokyo, Japan) operating at 200 kV and by Z-contrast imaging using a high-angle annular dark field (HAADF) detector in a JEOL 2010 FEG microscope working at 200 kV in scanning TEM (STEM) mode. Additionally, high-resolution STEM (HRSTEM) studies were performed using X-FEG FEI Titan microscope (FEI, Hillsboro, OR, USA) at 300 kV. This last microscope is equipped with a spherical aberration (C_s_) corrector for the electron probe (CEOS company, Heidelberg, Germany), allowing a probe size of 0.08 nm (mean size).

### Strain analysis

Maps of the strain along the growth direction (*ε*_zz_) were determined from HRTEM images acquired on the [110] pole axis using the geometrical phase analysis (GPA). The GPA is based on the calculation of the displacement field and subsequently the strain map by numerical derivatives, from the phase images for different and non-collinear vectors. A full description of the methodology can be found elsewhere [13]. The tensile strain along the (001) direction, *ε*_zz_, due to the tetragonal distortion in pseudomorphic samples under biaxial stress could be used to determine the in-plane strain *ε*_xx_ through the biaxial strain coefficient, *R*_B_ = −*ε*_zz_/*ε*_xx_, which in the case of cubic materials is equal to *R*_B_ = 2*C*_12_/*C*_11_, with *C*_*ij*_ being the Voigt values stiffness tensor. As GaAs and GaSb have the same biaxial strain coefficient (*R*_B_ = 0.899), the measurement of *ε*_xx_ allows us an assessment of the composition, assuming a compliance with Vegard's law at these contents.

### High-resolution Z-contrast analysis

The used method is based on the analysis of normalized integrated intensities (*R*) of high-resolution aberration-corrected Z-contrast images. The *R* values are calculated as the quotient of the integrated intensity around the cationic and anionic columns with respect to a binary compound (the substrate), which is chosen as a reference [12]. In our case, InAs QDs in GaAsSb(N) is a suitable system for Z-contrast analysis due to the difference in the atomic number of Sb (*Z*_Sb_=51) with respect to As (*Z*_As_=33), and In (*Z*_In_=49) with respect to Ga (*Z*_Ga_=31), and their almost linear dependency on the composition [9]. Firstly, local intensity maxima of each dumbbell are located by applying the peak detection tool of the Peak Pairs Analysis software [14]. Secondly, intensities from pixels corresponding to the cationic (In+Ga) and anionic (As+Sb) columns are integrated from the raw (unfiltered) image. From this point, we assume that the chemical information extracted from a single atomic column is weakly affected by the neighboring ones when using a sub-angstrom electron probe, and therefore, the signal is essentially related to the composition of the selected atomic column. In this case, two integrated intensity quotients (*R*_i_) were determined for every dumbbell in the image: *R*_1_, as the ratio between the integrated intensity in the anionic As/Sb column and the averaged integrated intensity of the As columns in the GaAs substrate within the same image; and *R*_2_ as the ratio between the integrated intensity in the cationic Ga/In column and the average integrated intensity of the Ga column in the GaAs region. The results of the *R*_*i*_ values are plotted over the HRSTEM image using a color scale where the reddest values are associated with a higher proportion of heavier elements, and the bluest, the contrary.

## Results and discussion

**Figure 1:**
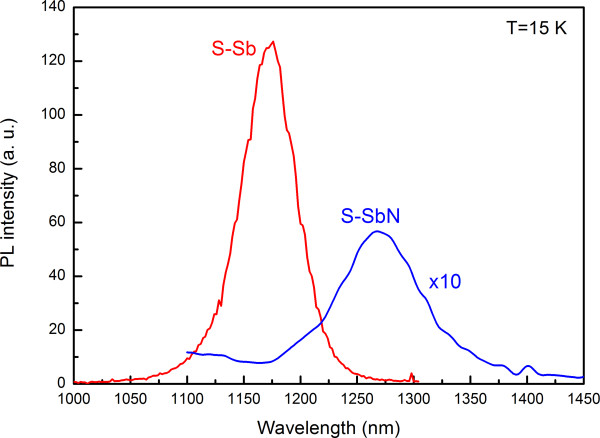
**15-K PL spectra of the two InAs/GaAs QD layers capped with GaAsSb and GaAsSbN.** The nominal Sb content is the same in both samples. The nominal N content in S-SbN is 2%.

Figure 1 shows the 15-K PL spectra of both samples. Adding N induced a redshift of 80 meV, but it also significantly increased the full width at half maximum (FWHM) and reduced the integrated intensity by a factor of 12. Although the large redshift can be partially explained by the reduced QD-barrier conduction band offset in the presence of N [7], nitrogen should also decrease the SRL redshift effect, the strong differences in the PL lineshape and intensity point to the presence of N-induced structural changes. In order to elucidate the possible reasons of this behavior, the samples were analyzed by transmission electron microscopy techniques to determine the strain fields and compositional distribution.

**Figure 2:**
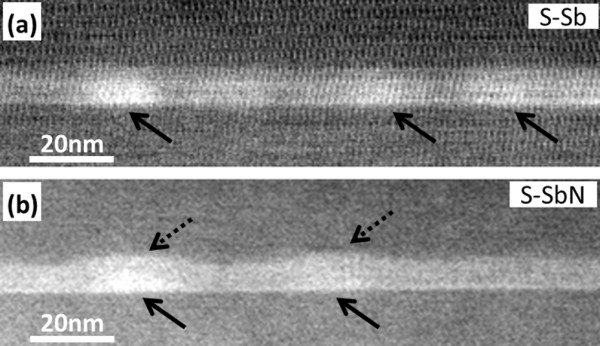
**HAADF images of samples (a) S-Sb and (b) S-SbN acquired at low magnification.** Dark solid arrows mark the QDs position, and dashed arrows in (**b**) show the GaAsSbN lobules formed over the QDs.

From CTEM analysis, no extended defects such as dislocations or planar defects were found in either of the samples (<10^8^ cm^−2^), denoting that the degradation of the PL efficiency by the addition of N should not be attributed to these kinds of defects. In Figure 2a,b, low-magnification HAADF-STEM images show representative views of samples S-Sb and S-SbN, respectively. In these Z-contrast images, regions with higher content of heavy elements appear brighter, thus making possible a clear distinction between the position and shape of the QDs and the CL surrounded by GaAs. After analyzing 75 QDs, the average QD base and height were measured as 13 ± 2 nm and 3.4 ± 0.6 nm in both samples, and no appreciable differences in the QD size are observed within the statistical error. The addition of N to the GaAsSb CL does not seem to influence the structure of the QD itself. However, this was not exactly the case for the CL. It should be noted that the CL in the region between QDs was thicker in the S-Sb sample (8.7 nm) than in the S-SbN (7.2 nm). Furthermore, sample S-Sb shows a planar top interface of the CL even when it wraps up the QD, that is to say, the CL over the QDs is thinner than in between QDs. A strong Sb segregation in the growth direction was observed in areas between QDs. By contrast, the CL of the S-SbN sample is adapted to the wetting layer QD surface, trying to keep the thickness constant, and therefore causing a growth front undulation when it covers the QDs. This is the first clue that the combination of N and Sb in the capping layer does not only lead to a band offset and a strain modification effect but also has a strong effect on the morphology of the structure.

**Figure 3:**
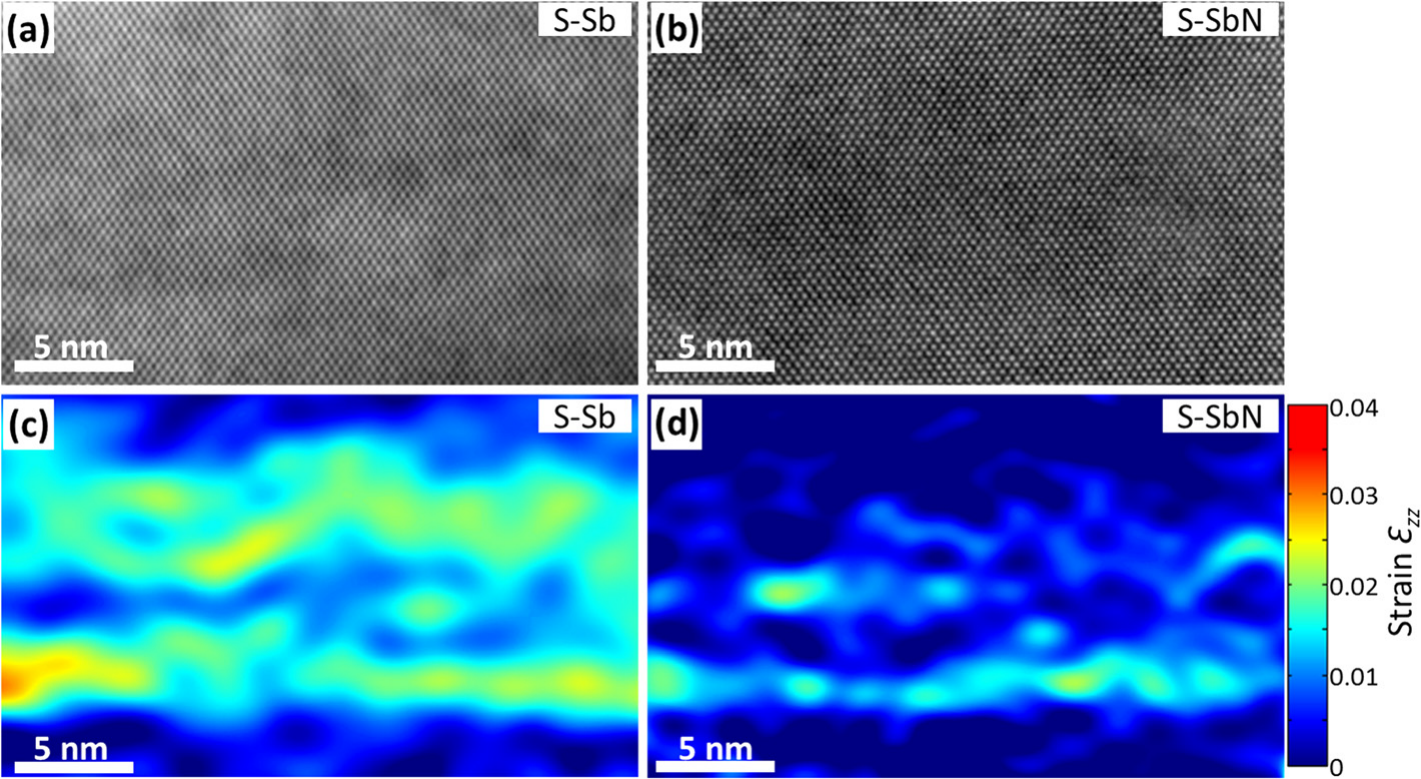
**HRTEM images and strain maps of CLs.** The images were acquired along the [110] zone axis of the CLs in the samples (**a**) S-Sb and (**b**) S-SbN, and (**c**,**d**) their strain maps, respectively. Color scales of the strain are shown on the right.

In order to evaluate the strain in the CL, HRTEM images were acquired on the [110] zone axis far from any QD (Figure 3a,b) and processed to obtain strain maps using GPA [13]. From the measurement of *ε*_*zz*_ and assuming the compliance with Vegard's law at these contents, the Sb composition in the CL of sample S-Sb was estimated. Thus, in the CL regions between QDs for sample S-Sb (Figure 3c), the average strain measured, *ε*_zz_ = 1.5 ± 0.2%, corresponds with a Sb content of 10.3 ± 0.4%, close to the designed value of 12%. In contrast, a reduced *ε*_zz_ = 0.6 ± 0.1% was observed in comparable areas of the S-SbN sample (Figure 3d). This large reduction needs to be attributed to N incorporation, which rises to 2.6 ± 0.4% (in reasonable agreement with the nominal composition of 2%). In addition, as is observed in Figure 3d, the strain distribution in the CL of the S-SbN sample becomes more inhomogeneous, which points to a composition modulation with the presence of Sb-rich and Sb-poor regions in the range of a few nanometers. Certainly, the addition of N enhances even more the large miscibility gap of GaAsSb [15] and gives rise to strong composition fluctuations. The observed clusters can act as traps for carrier, reducing the injection efficiency in the InAs QDs. The presence of the N-induced non-radiative centers (characteristic in N-diluted alloys) [16] together with the higher compositional inhomogeneities is a possible explanation for the degradation of the PL.

**Figure 4:**
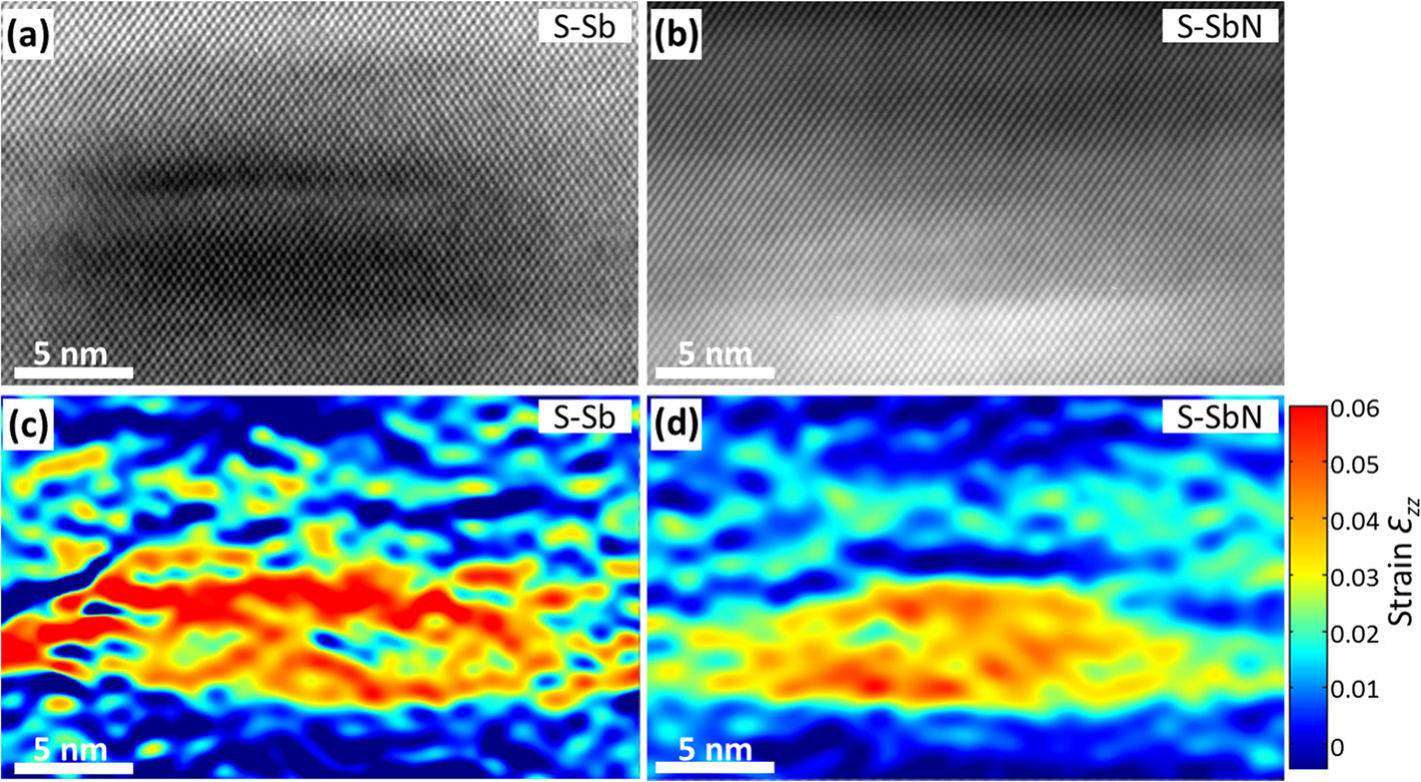
**HRTEM images and strain maps of QDs.** The images were acquired along the [110] zone axis of the QDs in the samples (**a**) S-Sb and (**b**) S-SbN, and (**c**,**d**) their strain maps, respectively. Color scales of the strain are shown on the right.

However, the situation changes in and around the QDs. Figure 4 shows the HRTEM and strain maps of QDs from both samples. Firstly, the average strain *ε*_zz_ evaluated within the core of up to five QDs of the S-Sb sample was 3.8 ± 0.2%, which is very similar to the average strain of 3.5 ± 0.2% measured in several QDs in S-SbN. Secondly, the average strain in the CL region above the QDs was also similar in both cases, with the *ε*_zz_ being 1.3 ± 0.2% and 1.5 ± 0.2% for the S-Sb and S-SbN samples, respectively. Important conclusions can be extracted from the strain data. The marked strain release in the CL between QDs when adding N (*ε*_zz_ lowers from 1.5% to 0.6% as mentioned in the previous paragraph) should entail a decrease in *ε*_zz_ inside the QD due to the reduced SRL effect [17]. This did not happen, and the explanation of the observed result implies that the CL must undergo clear changes in the surroundings of the QDs. Assuming a homogenous distribution of N over the CL in S-SbN, the measured strain implies that the Sb content in that region must be approximately 17%, which indicates a strong Sb migration towards the top of the QDs in the S-SbN sample. This assumption of a homogeneous N distribution is in agreement with the results of Ciatto et al. [18], who found that the Sb local environment is essentially random and that no significant preferential Sb-N pairing occurs over random statistics. The higher Sb content on top of the QDs compensates the counteracting effect of N on the strain reduction effect of the CL, explaining the similar strain values also observed inside the QDs. It seems that the system rearranges itself through composition modulation in the CL in order to keep the total strain in the QD region constant and equal to what would likely be the most stable value.

**Figure 5:**
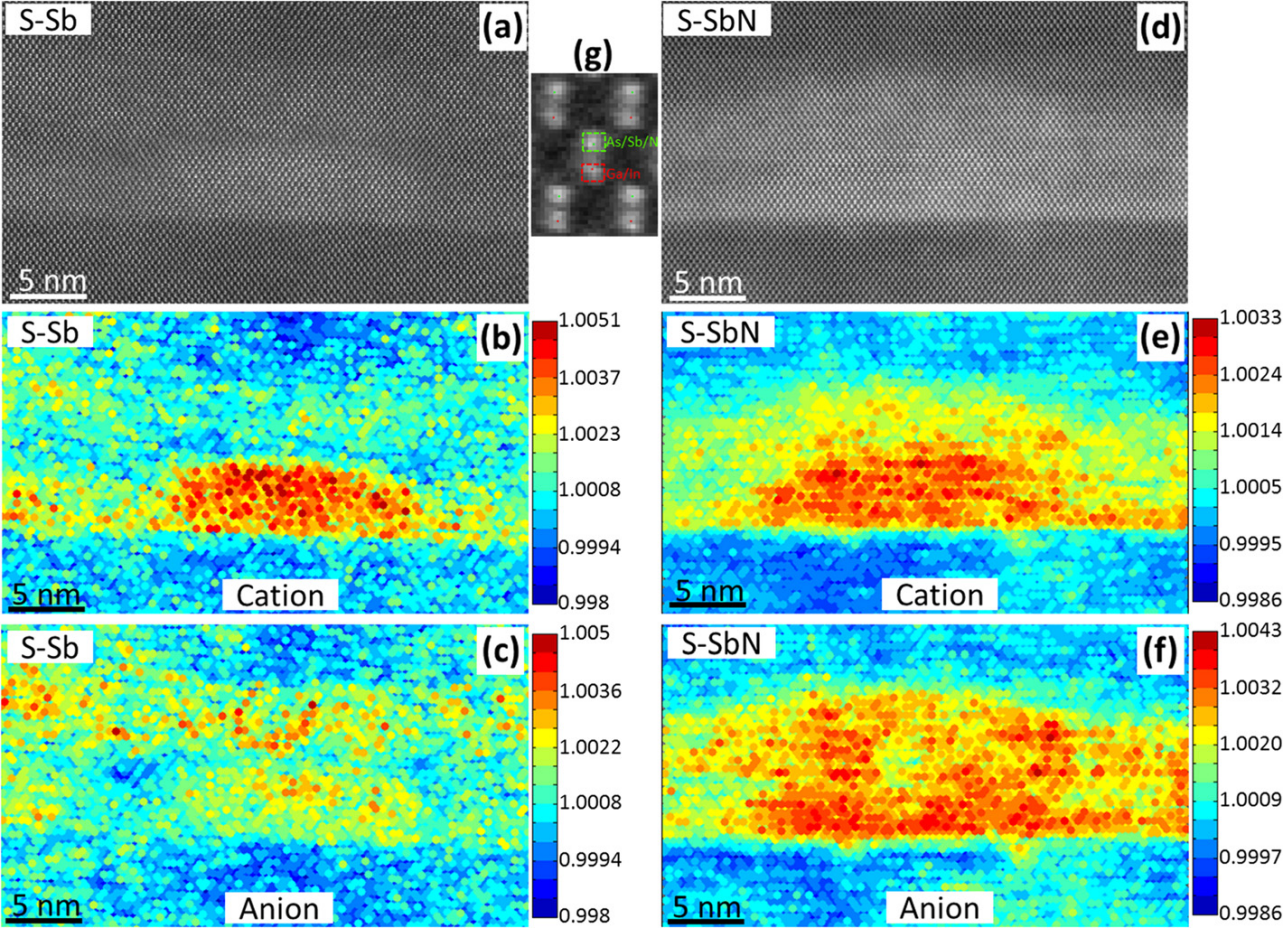
**HR Z-contrast images of QDs and their integrated intensity (compostition related) maps.** The images were acquired along the [110] zone axis of samples (**a**) S-Sb and (**d**) S-SbN, and their respective maps of the *R* values (*R*_*i*_) of (**b**,**e**) cations and (**c**,**f**) anions. Higher values (hot colors) can be attributed to atomic columns with heavier elements, In or Sb, respectively. (**g**) The regions used for intensity integration over each atomic column.

To confirm our hypothesis about the chemical composition in and around the QDs, HRSTEM Z-contrast imaging was carried out for both samples (Figure 5a,d). Anion and cation sub-nets are resolved in these images, showing the characteristic ‘dumbbells’ found at the [110] zone axis orientation. Figure 5g shows a magnified dumbbell where the upper and lower columns are the anionic and cationic components, respectively. In order to estimate column-by-column the Sb and In contents, a method similar to the one used in [9,19] was performed. The *R* values corresponding to each sub-net column, *R*_*i*_, are represented in Figure 5 with colored dots, where higher values (red dots) are associated with atomic columns with higher proportion of heavier elements with respect to the corresponding atomic columns in GaAs. It is assumed in this work that the minimum *R*_*i*_ measured in each image corresponds to a pure Ga or As column, respectively. Taking all this into account, Figure 5b,e depicts the estimated In/Ga distribution of both samples. Red dots, related to In-rich atomic columns, perfectly draw the QD and wetting layer (WL) regions. No appreciable out-diffused In from the QD was evinced. Still, in the case of the anionic positions, the panorama is very different when comparing the two samples. The redder dots in Figure 5c,f are associated to anionic columns with a higher content of Sb. As it is shown in Figure 5c for the S-Sb sample, the Sb atoms are mainly segregated to the upper region of the CL, leading to a Sb-poor region just above the WL. This is in agreement with previous results from scanning tunneling microscopy [5,20], which reported that a region depleted of both Sb and In is distinguishable at the WL-CL and QD-CL interfaces. This kind of behavior - the accumulation of Sb atoms on the growth-front surface - has been reported in many III-V systems [21,22], and it is a consequence of the bond energy differences. Since the Ga-Sb bond is considerably weaker than the Ga-As one, there is a preference for Sb atoms to be expelled to the surface [23]. Moreover, the Sb concentration in the CL of the S-Sb sample was not excessively altered by the presence of QDs. This could explain the lack of undulation on the growth front observed in the capping layer above the QD. Nevertheless, the Sb distribution intensely changes in the case of sample S-SbN (Figure 5f). In this case, the reddest dots were located on and around the QDs in a higher density than in sample S-Sb. The higher Sb content around the QDs agrees with the higher *ε*_zz_ values determined in the strain maps on the QD with respect to the CL regions in between the QDs that pointed to a lateral migration of Sb towards the QDs.

Certainly, the presence of N (with *Z* = 7) should give rise to a small decrease of the anionic *R*_1_ quotient. However, the number of anionic columns with *R*_1_ > 1 in S-SbN amply exceeds the observed ones in sample S-Sb. In this sense, statistical analysis in dilute nitrides of GaAsN had shown that the incorporation of N at low contents causes negligible changes in the brightness of the atomic columns but a strong increase in the valleys between them [24]. This fact does not affect our measurements since the pixels selected to calculate the *R* quotients just avoid the area between atomic columns [9]. However, the impossibility to detect the position and content of N around the QD, together with the non-negligible influence of static atomic displacements in the Z-signal, disables any attempt to quantify the Sb concentration in sample S-SbN by this technique [25].

All these results indicate that the addition of N to the GaAsSb capping layer increases the amount of Sb on top and around the QDs, inducing an undulation in the capping layer which tends to adapt to the morphology of the QD below it. Though this migration leads to an enrichment of Sb around the QDs, the strain-compensating effect of N gives rise to similar strain fields around and inside of the S-SbN QDs to those for the S-Sb ones. When the CL covers a QD, nitrogen fosters the Sb to accumulate on top of the partially relaxed InAs QDs since GaSb has a very similar lattice constant with InAs. On the other hand, as the composition threshold for the total restraint of GaAsSb/InAs QD dissolution in the capping process is around 11% to 14% of Sb [26,27], we could assume that the dissolution process of InAs QDs during the capping growth is completely suppressed for both samples and that the In atoms are not being relocated from the top of the QDs to the QD base [1,7,28]. This explains why no significant differences in the size and morphology of the QDs are seen in both samples. The addition of N greatly enhances the lateral Sb segregation, and this fact could even induce to a transition to a type II band alignment in the VB, which is expected for Sb contents of 14% to 17% [1,28]. Further work is in progress to clarify this issue.

## Conclusions

In summary, we have presented PL results and compositional distribution analysis of two InAs/GaAs QD samples capped by GaAsSb SRL with and without N incorporation. First, the addition of N produces a long redshift in the PL spectra, but it also reduces its efficiency by increasing the FWHM and reducing the intensity. However, the TEM results show that the PL behavior is not due to modifications in the QD sizes or to an increase in the extended defect density. On the other hand, the deformation analysis displays important changes in the nanostructure when N is added: (1) a large strain reduction in the CL regions away from the QDs and (2) an enhancement of the miscibility gap that gives rise to strong composition fluctuations and reduces the carrier injection efficiency in the InAs QDs. On the contrary, the average strain within the core of QDs and in the CL region above the QDs was also similar in both cases, which suggests a strong Sb migration towards the top of the QDs in the S-SbN sample (approximately 17%). Certainly, compositional maps of column resolution from aberration-corrected HAADF HRSTEM images corroborated the preferential deposition of Sb above the InAs QD together with an undulation of the growth front by the addition of N. Together with the reduction of the CB offset by the N incorporation, an additional redshift is induced due to a Sb accumulation on top of the QDs. Therefore, the N implementation could boost the features of GaAsSb capping layers on InAs QDs since it multiplies the Sb content around the QDs.

## Competing interests

The authors declare that they have no competing interests.

## Authors’ contributions

JMU and AH designed and grew the samples and carried out the PL study. DFR acquired TEM data and carried out the analysis of results. LD prepared the TEM samples and, together with AM, collaborated in the TEM data acquisition. DG and DS designed the TEM studies and supervised the TEM analysis. All authors actively discussed the results and participated on drafting the manuscript, and they all read and approved the final one.
